# Morphological study of the integument and corporal skeletal muscles of two psammophilous members of Scincidae (*Scincus scincus* and *Eumeces schneideri*)

**DOI:** 10.1002/jmor.21298

**Published:** 2020-11-09

**Authors:** Jérôme Canei, Denis Nonclercq

**Affiliations:** ^1^ Laboratory of Histology, Biosciences Institute, Faculty of Medicine and Pharmacy University of Mons Mons Belgium

**Keywords:** skinks, sand dwelling, muscle fibers, integument, immunohistochemistry

## Abstract

Sand deserts are common biotopes on the earth's surface. Numerous morphological and physiological adaptations have appeared to cope with the peculiar conditions imposed by sandy substrates, such as abrasion, mechanical resistance and the potential low oxygen levels. The psammophilous scincids (Lepidosauria) *Scincus scincus* and *Eumeces schneideri* are among those. *S*. *scincus* is a species frequently used to study displacement inside a sandy substrate. *E*. *schneideri* is a species phylogenetically closely related to *S*. *scincus* with a similar lifestyle. The aims of this study focus on the morphology of the integument and the muscular system. Briefly, we describe interspecific differences at the superficial architecture of the scales pattern and the thickness of the integument. We highlight a high cellular turnover rate at the level of the basal germinal layer of the epidermis, which, we suggest, corresponds to an adaptation to cutaneous wear caused by abrasion. We demonstrate the presence of numerous cutaneous holocrine glands whose secretion probably plays a role in the flow of sand along the integument. Several strata of osteoderms strengthen the skin. We characterize the corporal (*M*. *longissimus dorsi* and *M*. *rectus abdominus*) and caudal muscular fibers using immunohistochemistry, and quantify them using morphometry. The musculature exhibits a high proportion of glycolytic fast fibers that allow rapid burying and are well adapted to this mechanically resistant and oxygen‐poor substrate. Oxidative slow fibers are low in abundance, less than 10% in *S*. *scincus*, but a little higher in *E*. *schneideri*.

## Graphical abstract

Photograph of holocrine glands between the folds of the skin of *Scincus scincus*. Compound osteoderms are located on both sides. The skin surface is visible on the upper right side.

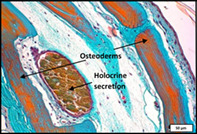

## 1 INTRODUCTION

Sand is, by definition, a granular material, finer than gravel and coarser than silt. Sandy substrates are present in terrestrial, lacustrine, and marine environments, and cover a large part of the surface of our planet, for example, 20% of deserts are sandy (Seely, 1991). Organisms living in sand must deal with the impact of the physical properties of this material, such as abrasion due to the constant friction between the body's surface and sand grains (Klein, Deuschle, & Gorb, 2010). Sandy substrates also induce oxygen depletion due to limited air diffusion (Vihar, Wolf, Böhme, Fiedler, & Baumgartner, 2015). The soil gas diffusion coefficient and its dependency on air‐filled porosity govern most gas diffusion processes in soil. This diffusion depends on numerous physicochemical parameters of the substrate, such as its granulometry, the composition of the sand, its hydration, its temperature, and the partial pressure of O_2_ (Moldrup, Olesen, Yoshikawa, Komatsu, & Rolston, 2004). Mechanical resistance, beside abrasion and hypoxia, constitutes a third parameter of major importance, which can induce high energy expenditure (Seymour, Withers, & Weathers, 1998). The energetic cost of swimming through sand has been evaluated for a psammophilous mammal and the energy expenditure for sand swimming was 26 times more expensive when compared to surface running (Seymour et al., 1998). Psammophilous species have developed morphological, physiological, and behavioral adaptations to cope with sandy substrates conditions (Etheridge, 2000). Many sand dwelling lizards, such as *Uma scoparia* (Carothers, 1986) have developed fringes on their digits to enable locomotion. Some lizards exhibit a high degree of behavioral adaptation, such as the gerrhosaurid *Angolosaurus skoogi*, which is considered a “sand swimmer” because of its capacity to move through the sand by fluidizing it (Baumgartner et al., 2008). Other families of lizards include species adapted to burrowing in sand, such as *Callisaurus draconoides* and *Agama etoshae*. Some species of psammophilous geckos have reduced their toe pads and exhibit spinose digital scales (Lamb & Bauer, 2006). Moreover, the composition of sand as expressed in the presence of different grain sizes seems to have an impact on the spatial distribution of lizard species and on the territorial distribution of metapopulations within species, as for *Sceloporus arenicolus* (Ryberg & Fitzgerald, 2015; Ryberg, Hill, Painter, & Fitzgerald, 2013). In some hypogeal species of lizards, such as *Scincus scincus*, physiological adaptations of the nasal cavity and respiratory tract prevent sand grains from entering the lungs (Stadler et al., 2016). Here, we investigate two species of Lepidosauria, *S*. *scincus* (Linnaeus, 1758) and *Eumeces schneideri* (Daudin, 1802). These two scincids have an overlapping geographical distribution, including the Arabian Peninsula and North Africa (Arnold & Leviton, 1977; Ayaz, Çiçek, Tok, & Dinçaslan, 2011) and are phylogenetically closely related (Pyron, Burbrink, & Wiens, 2013). The omnivorous sandfish (*S*. *scincu*s) is able to detect its prey, mainly insects, by vibratory cues (Hetherington, 1989) and lives in hot deserts supporting only sparsely distributed dry shrubs. The omnivorous Berber skink (*E*. *schneideri)* also lives in similar biotopes, but with relatively dense vegetation (Allam, Abo‐Eleneen, & Othman, 2017). These two skinks share a common behavioral ecology by living in sand, almost all the time for *S*. *scincus* (Stadler et al., 2016) and at least for some part of the day for *E*. *schneideri* (Saleh, 1997).

*S*. *scincus* is used as a model species in kinematic and biophysical studies focusing on the efficient subsurface “swimming” locomotion mechanism of this species (Arnold, 1995; Baumgartner et al., 2008; Dorgan, 2015; Knight, 2012; Maladen, Ding, Li, & Goldman, 2009; Maladen, Ding, Umbanhowar, & Goldman, 2011; Maladen, Umbanhowar, Ding, Masse, & Goldman, 2011; Sharpe et al., 2015; Sharpe, Ding, & Goldman, 2013). The neuromechanical strategy of *S*. *sincus* during walking, burial, and swimming inside the substrate was also investigated (Sharpe et al., 2013). This species is also well‐known for numerous morphological adaptations to sandy substrates including a shovel‐shaped snout, a subquadrangular cross section, small eyes, reduced ear openings, and fringes present on its digits (Baumgartner et al., 2008). Here, we focus on the morphology of the integument and the muscular system, poorly investigated in *S*. *sincus* and never in *E*. *schneideri*. The shape of the scales, the epidermal turn‐over, the rigidity of the skin linked to the presence of osteoderms, the presence of cutaneous secretions are important morphological characteristics which can limit the frictions of the substrate or form points of support for the musculature. The composition of the musculature (relative proportion of slow, fast, and intermediate fibers) is also an important factor for displacement in a largely anaerobic and mechanically resistant environment. All of these different parameters will be analyzed and discussed in this study. These organs observed in *S*. *Scincus* have been compared with the related species *E*. *schneideri*, which is used in the present study as a partial control considering the fact that this species is less dependent on subterranean life. We hypothesize that the integument of *E*. *schneideri* is less thick and resistant to abrasion and a corporal musculature with a higher percentage of oxidative slow fibers associated with a more aerobic environment. These morphological characteristics will be examined in a comparative study of both species.

## 2 MATERIALS AND METHODS

### 2.1 Animals

**FIGURE 1:**
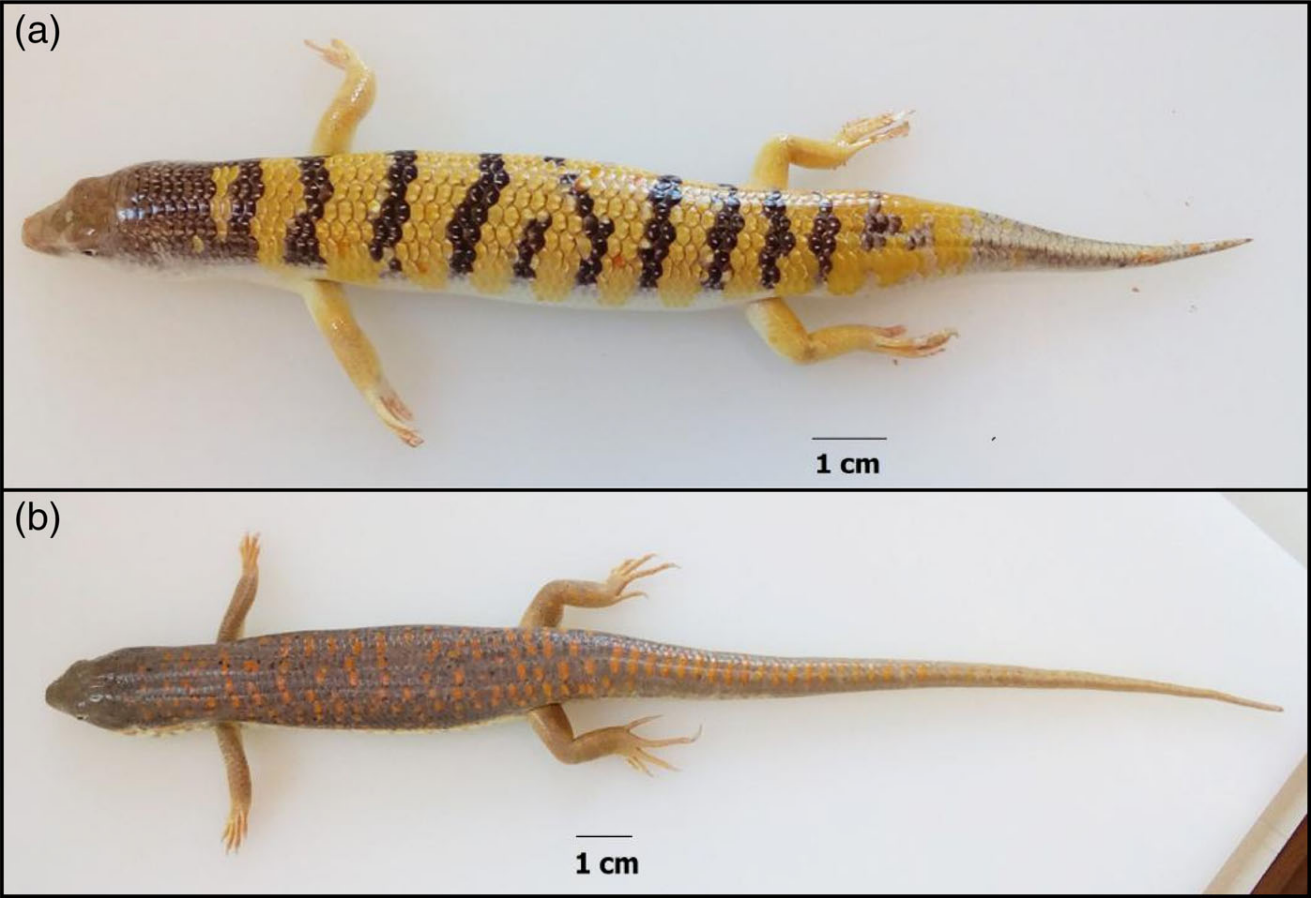
Dorsal views of *Scincus scincus* (a) and *Eumeces schneideri* (b)

Two males and one female (mean snout–tail length [STL]: 16.25 cm ± 2 mm) of *S*. *scincus* (Linnaeus, 1758) (Figure 1a) were used for histological analysis. These specimens were obtained from the Animal House “Animalerie 2000” Mouscron, Belgium. Three adult males (mean STL: 32.66 cm ± 2 mm) of *E*. *schneideri* (Daudin, 1802) (Figure 1b) were also used. Those specimens were obtained from the Animal House “Reptiles Univers” Dour, Belgium. All animals were maintained in the animal housing facility of the University of Mons (UMONS, Belgium). The animals were treated according to the guidelines specified by the Belgian Ministry of Trade and Agriculture and under the control of the UMONS ethical commission (agreement LA1500021).The lizards were housed individually in terraria (120 × 60 × 60 cm), the bottom of which was covered with a layer of fine sand 10–12 cm deep (with a particle diameter comprised between 0.75 and 1.3 mm). Each terrarium was equipped with a UV lamp (5.0 UVB, 26 W; ZooMed) and an infrared bulb for heating (Ceramic infrared, 60 W; ZooMed). Both light bulbs were placed at a height of 35 cm. The animals were maintained in a controlled environment with 12 hr of daylight and a temperature of 35°C during the day and 22°C at night. The animals were fed with live adult house crickets (*Acheta domesticus*), and vegetables twice a week and received water ad libitum. All animals were euthanized (under the guideline of the UMONS Committee for Survey of Experimental Studies and Animal Welfare) by a lethal intramuscular injection of ketamine hydrochloride (200 mg/kg body mass, Ketalar). Each subject was considered dead when it lost the ability to respond to foot and tail pinching.

### 2.2 Histology

Following euthanasia, all individuals were cut transversely into 1 cm thick slices, and these slices were fixed by immersion in Duboscq–Brazil fluid (composition: formalin/acetic acid/ethanol containing 1% picric acid/distilled water, respective proportions: 260/70/425/245 by vol) for 48 hr and decalcified in 5% trichloroacetic acid for 48 hr. Following dehydration and paraffin embedding, the blocks were cut in serial sections of 5 μm thickness on a Leica RM 2145 microtome and placed on silane‐coated glass slides. After rehydration, the first sections of each consecutive series of slides were stained with hematoxylin, Orange G, and Fast green (Masson's Trichrome stain). The following successive sections of each series were used for immunohistochemical procedures described in the following paragraph. The sections were observed on a research optical microscope (Leitz Orthoplan) equipped with a high sensitivity camera (Leica DFT7000 T, Germany) and pictures were recorded using Leica Application Suite X, LAS X, Germany software.

### 2.3 Immunohistochemistry

#### 2.3.1 Identifications of muscle fiber types

Serial sections of the body of *S*. *scincus* and *E*. *schneideri* individuals were used to study muscle fiber typing. The body muscles targeted (see Sharpe et al., 2013) were the *M*. *longissimus dorsi* for the dorsal parts, and the *M*. *rectus abdominus* for the ventral parts (supplementary online material, Figure S1a). The muscular bundles located beyond the cloaca were considered as “tail muscles.” The precise nomenclature of the tail muscles is not clearly specified. Consequently, we have chosen to consider all the muscular fibers surrounding the tail vertebras as indicated on (supplementary online material, Figure S1b). Along the animal's body and tail, at regular intervals of 1 cm, two consecutive sections of 5 μm thick were taken and treated for immunostaining of fast and slow muscle fibers, respectively. This allowed us to compare the distribution of the different fiber types in consecutive sections. Prior to immunostaining, dewaxed sections were rehydrated and rinsed in distilled water. The unmasking of antigenic sites was performed by microwave heating in 0.01 mol L^−1^ citrate buffer (pH = 6.2). Sections microwaved in two consecutive runs (±5 min) at a power of 900 W, separated by a 15 min break. Heating was stopped when the first boiling bubbles appeared. After rinsing in distilled water, endogenous peroxidase activity was quenched by a 5 min exposure to 0.5% H_2_O_2_. Before applying the primary antibodies, the pretreated sections were rinsed in PBS (0.04 mol L^−1^ Na_2_HPO_4_.12H₂O, 0.01 mol L^−1^ KH_2_PO_4_, and 0.12 mol L^−1^ NaCl, pH 7.4) and incubated in 0.5% casein (diluted in PBS) for 15 min. Tissue sections were incubated with a monoclonal antibody raised against fast myosin (MY‐32) or with an anti‐slow myosin monoclonal antibody (NOQ7.5.4D). Both commercial monoclonal antibodies were raised in mice and purchased from Abcam (Cambridge, UK). These primary antibodies were used at their optimal working dilution (1:50) and histological sections were incubated for 1 hr at room temperature. After rinsing in PBS, slices were treated with the complex ImmPRESS HRP/Anti‐MouseIgG/Polymer (Vector, Burlingame, CA) for 30 min at room temperature. Bound peroxidase activity was visualized by precipitation of 3.3′ diaminobenzidine (DAB) 0.02% in PBS containing 0.01% H_2_O_2_. Preparations were counterstained with hemalun and luxol fast blue, dehydrated and mounted with a permanent medium. Controls for the specificity of immunolabeling included the omission of the primary antibody or the substitution of nonimmune sera for the primary antibodies. In each case, these controls were negative.

#### 2.3.2 Cell proliferation

Proliferating cells were detected using a monoclonal antibody raised against proliferating cell nuclear antigen (PCNA), the accessory protein associated with DNA‐polymerase δ. This method has been detailed in a previous publication (Piron et al., 1998). Briefly, serial sections of the body of *S*. *scincus* and *E*. *schneideri* were used to study the proliferation of epidermal cells. At each centimeter along the lizard body, a 5 μm thick section was used for immunohistochemistry. The immunostaining method is similar to that used for muscle fiber typing (see above), except for the unmasking of antigenic sites. Sections were immersed in a Pyrex container filled with distilled water and placed in a pressure cooker container with 750 ml distilled water at the bottom. Once firmly closed, the sections were submitted to high pressure at 120°C. After 20 min, the pressure cooker was turned off, and the sections were cooled at room temperature for 30 min. The sections were then immersed in distilled water, H₂O₂, PBS, and casein, as explained previously. The primary antibody used was the mouse monoclonal (PC10) anti‐PCNA from Abcam at its optimal working dilution (1:50). This antibody targets PCNA protein (also called DNA‐polymerase δ accessory protein) which plays a key role in DNA replication. The last part of the protocol is similar to the previous one detailed for muscle fiber typing. Controls for the specificity of immunolabeling included the omission of the primary antibody or the substitution of nonimmune mouse serum for the primary antibody. In each case, these controls were negative.

### 2.4 Morphometry

#### 2.4.1 Muscle fibers

For each species, the number of fibers and the area occupied by each type of muscle fibers was quantified by morphometric analysis at ×200 magnification. The procedure was based on a hardware system consisting of a Zeiss Axioplan microscope equipped with a ProgRes C10 plus color camera (Jenoptik, Germany) connected to an IBM‐compatible PC, and using software designed for morphometry and color analysis (KS 400 Imaging system, Carl Zeiss Vision GmbH, München, Germany). This image‐analyzing system discriminated between immunoreactive muscle fibers based on differences in color and contrast. For each image, eight standardized microscopic fields of 97.2 μm^2^ each were selected at random separately in the dorsal and ventral muscular layers. For each lizard (*n* = 3 per species), the absolute and relative areas occupied by the three categories of muscle fibers were calculated in the ventral and dorsal parts, as well as in the body and tail parts. Each measure was performed in triplicate on three consecutive histological slides. For each type of muscle fiber, the area of the fibers was divided by the total number of fibers to estimate the mean individual area of each type of fiber in cross sectional view in the dorsal and ventral parts (and also in the body and tail areas).

#### 2.4.2 Thickness of the integument

For the absolute thickness of epidermis and dermis, pictures were captured on an optical microscope (Leitz Orthoplan) equipped with a high sensitivity camera (Leica DFT7000 T, Germany) using specialized software (Leica Application Suite X, LAS X). The dorsal and ventral part of the body was measured separately in three specimens per species. All thickness measurements were done using ImageJ software (Schneider, Rasband, & Eliceiri, 2012). The relative thickness of integument was also calculated in both species. These values were obtained from absolute thicknesses values corrected relating to differences in overall body size between the species.

#### 2.4.3 Measure of cell proliferation

For each lizard (*n* = 3 per species), the number of epidermal proliferating cells was counted in 16 microscopic fields picked at random per slide at high magnification (×400). The area of each field was 0.084 mm^2^. These measurements were performed on transverse sections spaced every 1 cm along the animal to provide equal coverage from head to tail. The total number of nuclei (PCNA + and PCNA−) in the germinal layer of the epidermis was calculated for each species and corresponds to a mean of 22 total nuclei/microscopic field for *S*. *scincus* and 24 for *E*. *schneideri*. The proliferation index (expressed in %) is calculated by the ratio: number of PCNA positive nuclei per field/number of total nuclei per field.

#### 2.4.4 Scanning electron microscopy

Six small samples of the integument (<1 cm^2^) were collected dorsally (3) and ventrally (3) for each species. These were fixed in Duboscq‐Brazil's fluid for 24 hr. The fixed samples were then dehydrated in graded concentrations of ethanol and critical‐point‐dried using CO_2_ as the transition fluid. They were then mounted on aluminum stubs and coated with gold in a JEOL JFC‐1100E sputter coater. Images were obtained using a JEOL JSM‐7200F scanning electron microscope. The mean distance (width) between two consecutive microridges was measured using ImageJ software (Schneider et al., 2012). The values were compiled for the dorsal and ventral regions for both species. An estimation of the microridges height was also performed at the dorsal scale level.

### 2.5 Statistical analysis

All statistical analyses were conducted using the software for statistical computing R version 3.2.1, and the package pgirmess (Giraudoux, 2016). With respect to their distribution, the quantitative data obtained in this study were submitted to parametric (analysis of variance one‐way or Student's *t*) or nonparametric (Kruskal–Wallis or Wilcoxon) tests. *p*‐Values lower than .01 are indicated on the graphs by “<.01,” and those lying between .01 and .05 by “<.05.”

## 3 RESULTS

### 3.1 Histological analyses

**FIGURE 2:**
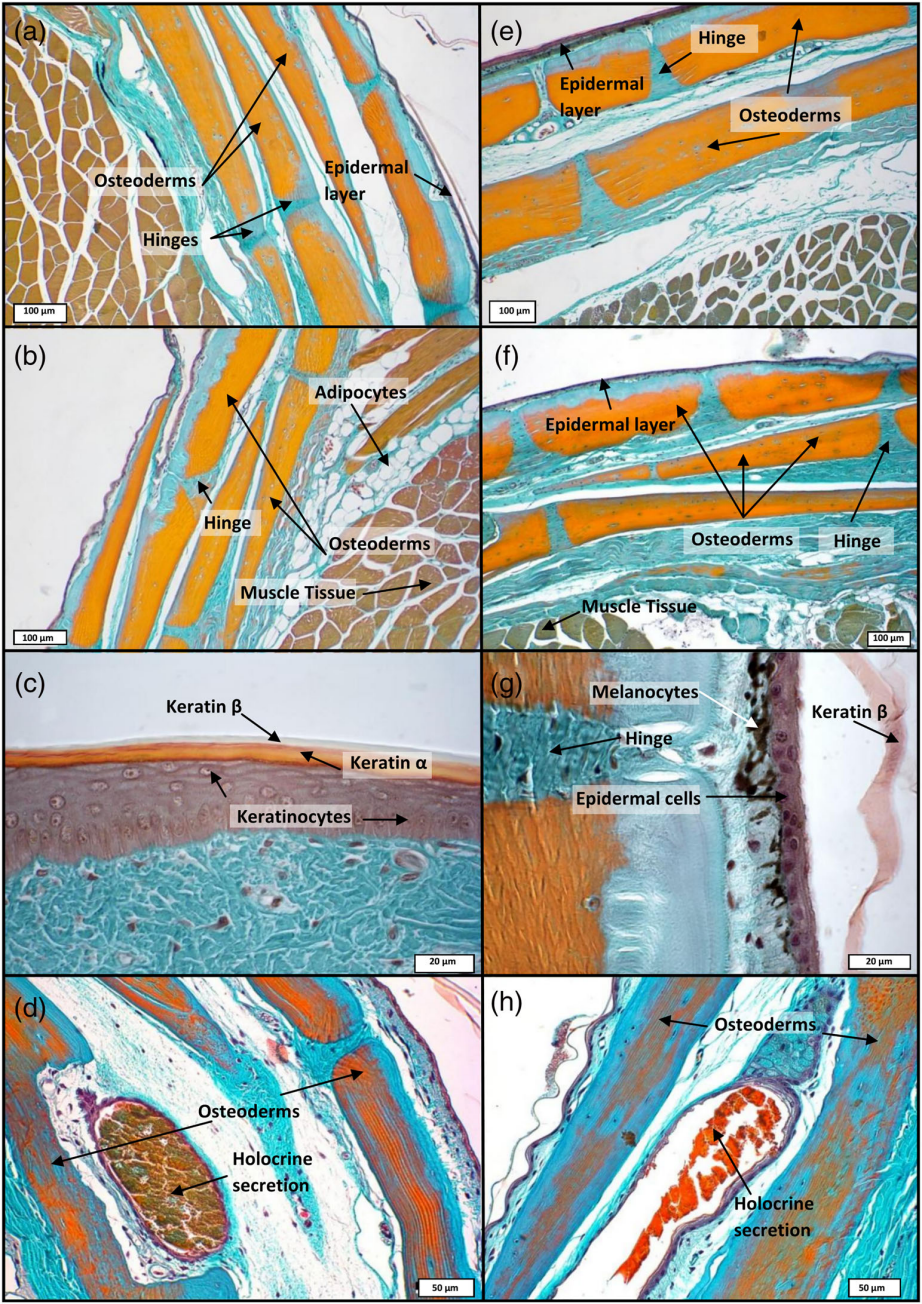
Integumentary morphology of *Scincus scincus* (left columns a–d) and *Eumeces schneideri* (right column e–h). All tissue sections were stained with Masson's Trichrome. (a,e) Low magnification images are from the body's dorsal region, whereas (b and f) illustrate sections from the body's ventral region. Numerous osteoderm layers, stained in orange, are present in the dermis of both species. Successive rigid osteoderm plates are articulated by hinges consisting of dense connective tissue. High magnification images (c and g) depict the epidermal layer of both species. The superficial alpha and beta keratin layers constituting the scale are clearly visible on (c), whereas (g) illustrates the presence of melanocytes in the superficial dermal stratum laxum. (d,h) The presence of holocrine glands between the folds of the skin superficial layers of holocrine glands in the junction between the folds of the skin superficial layers

**FIGURE 3:**
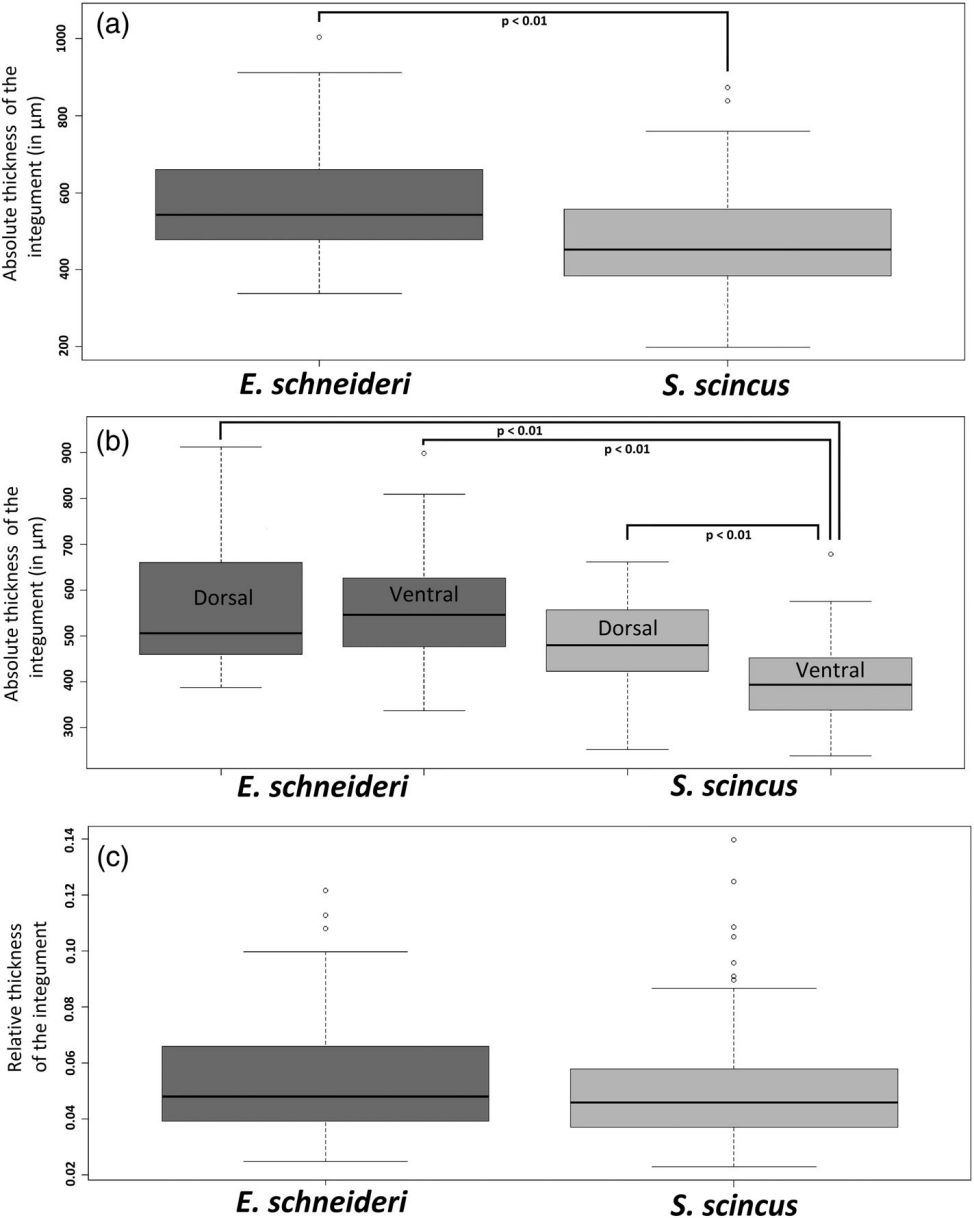
(a) Boxplots representing the absolute thickness (in μm) of the body integument (epidermal and dermal layers including osteodermic stratum) of *Eumeces schneideri* and *Scincus scincus*. (b) Similar boxplots representing the skin in absolute thickness (in μm) but assessed separately for the dorsal and ventral regions of *E*. *schneideri* and *S*. *scincus*. (c) Boxplots representing the relative thickness of the body integument (epidermal and dermal layers including osteodermic strates) of *Eumeces schneideri* and *Scincus scincus*. These relative values were obtained from absolute thicknesses values corrected relating to differences in overall body size between the species. Data were analyzed using the nonparametric Wilcoxon test (for (a and c)) and Kruskal–Wallis test (for (b)) and significant difference was set at a *p*‐value <.01

**FIGURE 4:**
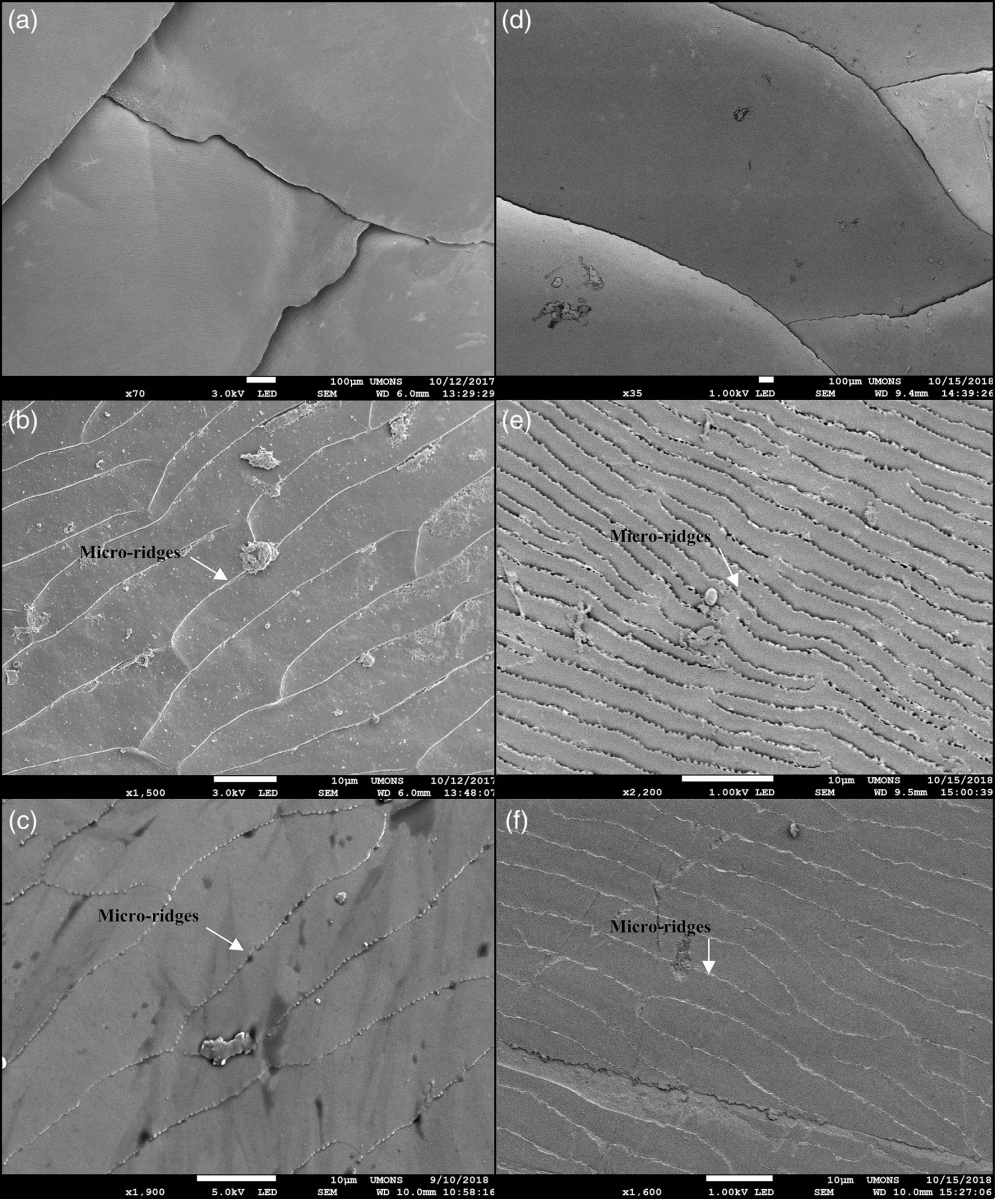
Scanning electron micrographs of scales of *Scincus scincus* (left column a–c) and *Eumeces schneideri* (right column d–f). (a,b) Low magnification images illustrate the imbrication of the dorsal body scales of both species. Images (b and e), taken at high magnification, indicate the micro relief present on the surface of dorsal scales, whereas (c and f) focus on the microridges of the ventral scales. The white arrows indicate microridges

**FIGURE 5:**
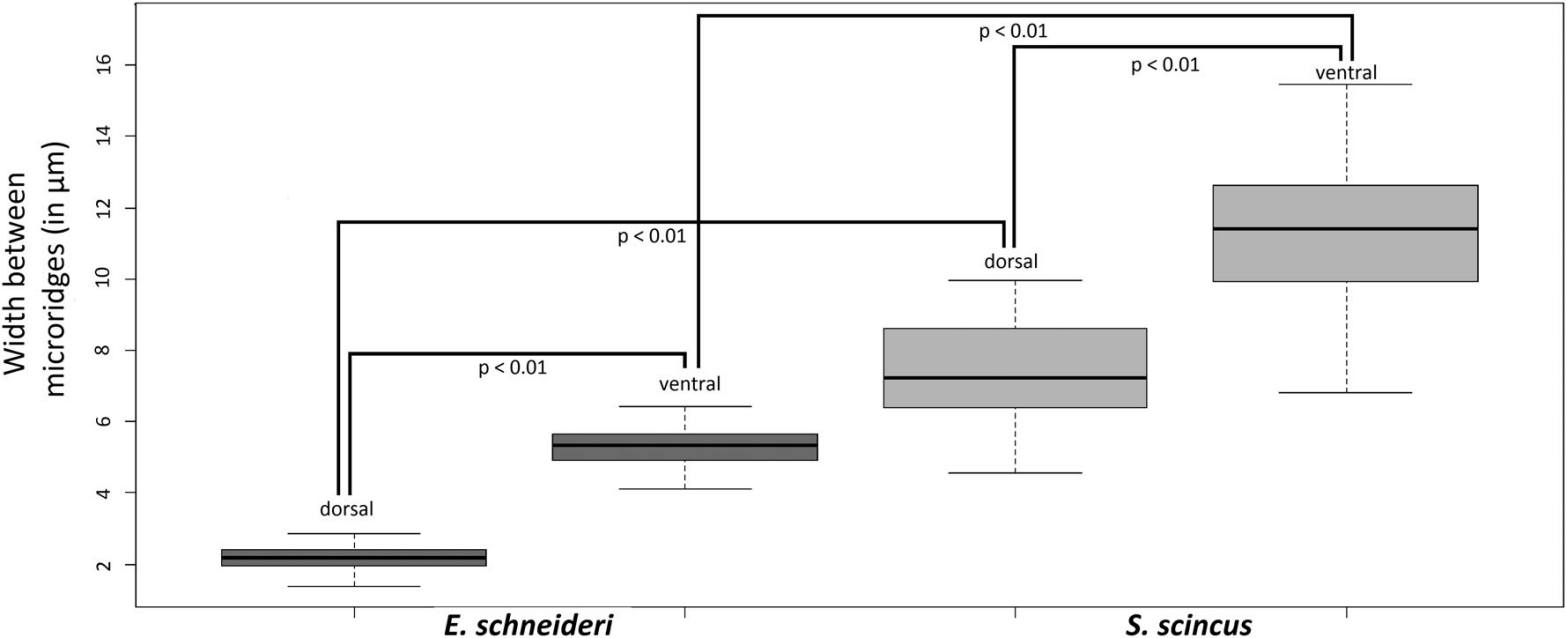
Boxplots representing the inter microridge spaces (in μm) of the scales of *Eumeces schneideri* and *Scincus scincus*. The measurements were taken from 9 scales (30 microridge values per body region) selected at random from the ventral and dorsal regions of both species. Data were analyzed using the nonparametric Kruskal–Wallis test and significant differences were set at a *p*‐value <.01

The integument (epidermis‐dermis) of both skink species was stained using Trichrome staining, allowing unambiguous identification of the different tissue layers. *S*. *scincus* (Figure 2a–d) and *E*. *schneideri* (Figure 2e–h) have a multilayered integument that incorporates stacked osteoderms (two to six layers for both species). These rigid features are present in all regions of the body, with their shape changing according to their location. The osteoderm plates alternate at regular intervals with thin zones of dense regular connective tissue forming hinges that allow for the flexibility of the integument. In the dorsal region of the body, the deep dermis exhibits at least two layers of superimposed osteoderms (Figure 2a,e). The two superficial strata are partially imbricating in the manner of roof tiles (Figure 2b). The deepest osteoderm layer consists of thicker and closer lamellar bone pieces. The osteoderms are separated from each other by connective tissue hinges, formed by dense, oriented collagen fibers, allowing relative mobility. Deep in the dermis is a hypodermic layer of white adipocytes (Figure 2b). On the ventral region of the body, the number of osteoderm layers also ranges between two and six (Figure 2b,f). In the apical part of the epidermis, orange staining indicates the keratinous nature of the scale surface (Figure 2c), with a more reddish coloration indicating α keratin, a type of soft keratin located in the basal layer of reptiles' scales, as well as in the junction between adjacent scales. On the micrographs, the outmost layer is β keratin, a more rigid type of keratin, which appears as translucent on histological slides (Figure 2c). This β keratin layer appeared to be frequently detached from the subjacent α keratin layer because the hard β keratin was easily removable due to mechanic action of the knife used during microtomy (Figure 2g). The superficial layer of the dermis (*stratum laxum*) consists of highly vascularized loose connective tissue with numerous melanocytes in the dorsal region of the body, giving the specific coloration to the skin (Figure 2g). The overall thickness of the integument (epidermal and dermal layers) was measured for all regions of the body surface (dorsal, lateral, and ventral) of both species. The averaged values were compared using the Kruskal–Wallis test (Figure 3). The mean values for *S*. *scincus* and *E*. *schneideri* were 465.58 and 582.43 μm, respectively. The absolute thickness (Figure 3a) differed statistically between the two species. Additionally, we compared the thickness of the integument separately for the dorsal and ventral regions of both species. Three significant differences were observed between the dorsal and ventral parts of both species, the ventral integument of *S*. *scincus* being significantly thinner compared to that of the other regions (Figure 3b). The relative thickness of integument was also calculated in both species. These values, illustrated in Figure 3c, were obtained from absolute thicknesses values corrected for differences in overall body size between the species. In this latter case, no significant differences were observed between both species. Additionally, in both species, we observed holocrine glandular structures at the hinge region between superficial osteoderm layers (Figure 2d,h for *S*. *scincus* and *E*. *schneideri*, respectively). These glands seem more abundant and voluminous in the ventral side of both species. The stratified glandular cells present a progressive accumulation of lipid droplets in their cytoplasm. These cells die and release their secretions in spaces between macroscopic folds of the skin (supplementary online material, Figure S2a,b). Images obtained by scanning electron microscopy (Figure 4) provide details of the microrelief on the surface of the scales (the outermost layer visible here being the Oberhäutchen). At low magnification (Figure 4a,d), the surface of the scales appears smooth in both species. However, at higher magnification, the scales (*S*. *scincus* [Figure 4b,c] and *E*. *schneideri* [Figure 4e,f]) show numerous microridges revealing a specific polygonal pattern. The distance between two consecutive ridges (width) was different between the two species. This distance is statistically greater in *S*. *scincus* compared to *E*. *schneideri*. The distances between the microridges from dorsal (Figure 4b,e) and ventral scales (Figure 4c,f) were statistically different; the distance being greater at the ventral level for both species (Figure 5). The mean values for *S*. *scincus* were 7.44 and 11.32 μm (dorsal and ventral, respectively) compared to 2.15 μm (dorsal) and 5.32 μm (ventral) recorded for *E*. *schneideri*. The mean height of those ridges, estimated at the dorsal level, is about 0.4 μm for both species, and there is no significant difference (boxplot not shown) for this parameter (see supplementary online material, Table S1 for all related microridges values).

### 3.2 Labeling of dividing epidermal cells

**FIGURE 6:**
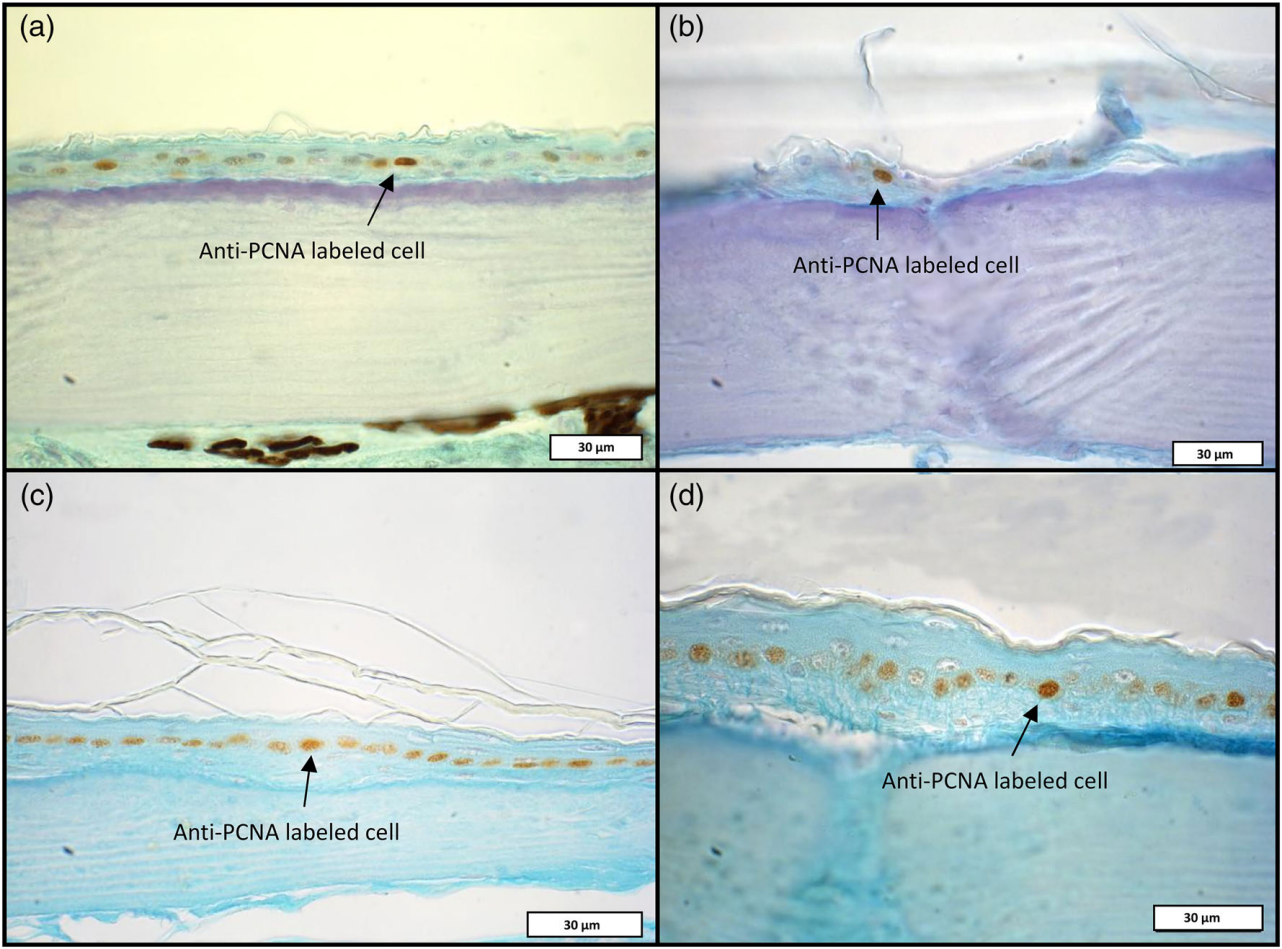
Immunohistochemical detection of dividing cells in the epidermal layer of *Scincus scincus* (a,b) and *Eumeces schneideri* (c,d). Anti‐proliferating cell nuclear antigen (PCNA) labeled cells are identified by a brown color in the nucleus (arrows)

**FIGURE 7:**
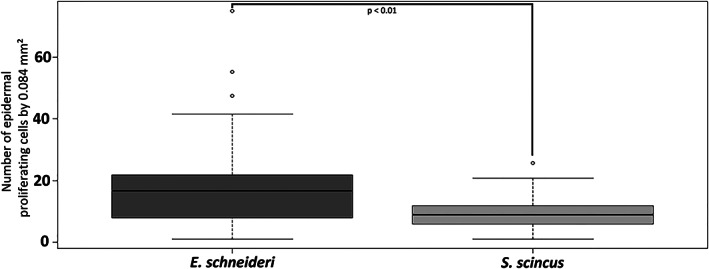
Number of epidermal dividing cells labeled with the anti‐proliferating cell nuclear antigen (PCNA) antibody per microscopic field of 0.084 mm^2^ for *Eumeces schneideri* and *Scincus scincus*, respectively. For each species (*n* = 3), 16 microscopic fields were selected at random from the head to the tail of each lizard. Data were pooled per species and presented as boxplots. Statistical analysis was performed using the nonparametric Wilcoxon test (significant differences was set at a *p*‐value <.01). The proliferation index is calculated by the ratio: number of PCNA positive nuclei per field/number of total nuclei per field and correspond to 37.7% for *Scincus scincus* and 64.7% for *Eumeces schneideri*

Proliferating epidermal cells were targeted in *S*. *scincus* (Figure 6a,b) and *E*. *schneideri* (Figure 6c,d) using anti‐PNCA antibodies. For both species, dividing cells were restricted to the basal regenerative layer of the epidermis. The number of dividing cells in the epidermal germinal layer is particularly important for both species, representing more than 50% of the basal cells for *E*. *schneideri*. Within each species, no significant difference in cells in S‐phase was observed between the dorsal and ventral body regions. The density of PCNA‐labeled cells appeared greater in *E*. *schneideri* than in *S*. *scincus*. To confirm this qualitative observation, we performed a morphometric analysis (Figure 7). The Wilcoxon‐test confirmed that cells in S‐phase were statistically higher in *E*. *schneideri* (*p*‐value < .01) with mean values of 8.29 labeled cells versus 15.53 labeled cells per microscopic field of 0.084 mm^2^ for *S*. *scincus* and *E*. *schneideri*, respectively.

### 3.3 Immunohistochemical typing of muscle fibers

#### 3.3.1 Muscle fibers

**FIGURE 8:**
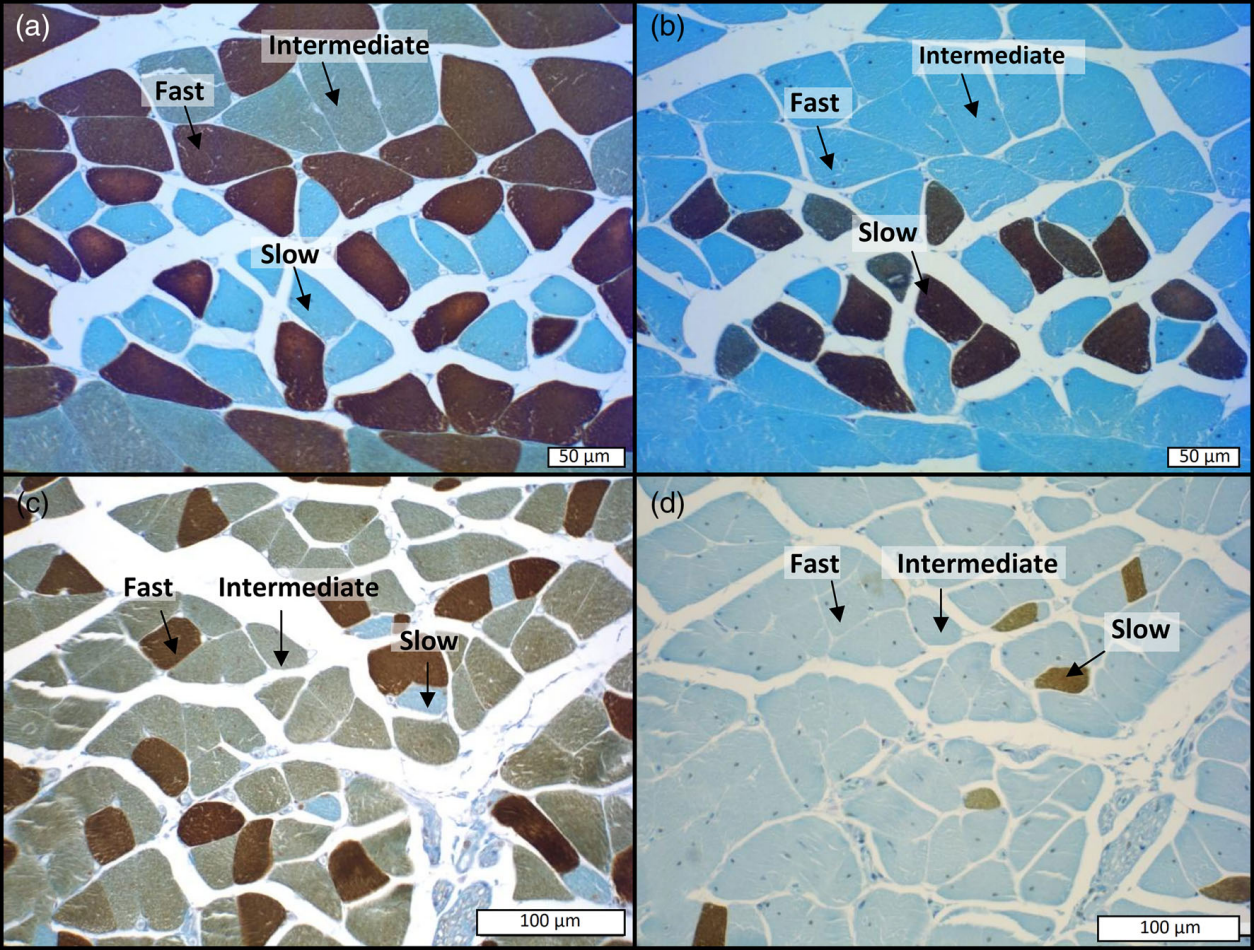
Illustration of the immunohistochemical method used for the typing of muscle fibers. The panel of four pictures corresponds to immunohistochemical labeling performed on serial sections of the muscles of *Scincus scincus* (a,b) and *Eumeces schneideri* (c,d), respectively. For each species, fast myosin was detected by a specific antibody (MY32 [Abcam]) in the first section (a,c) whereas slow muscle fibers were indicated in the following sections (b,d) by immunolabeling with specific anti‐slow myosin antibodies (NOQ 7.5.4D [Abcam]). On the left the pictures (a,c), correspond to the first section treated with antibodies raised against fast myosin, in which fast muscle fibers appear deeply stained in brown, intermediate fibers lightly stained in brown and slow muscle fibers negative (blue). In contrast, in the following sections (b,d), immunostained with antibodies raised against anti‐slow myosin only, slow muscle fibers are labeled in deep brown whereas intermediate and fast fibers are totally negative (blue color). The comparison between the consecutive sections (a vs. b and c vs. d) attests to the specificity of the immunolabeling and the clear‐cut identification of the three muscle fiber types

Three categories of muscle fibers (fast, slow, and intermediate) were identified on the basis of their immunoreactivity with the monoclonal antibody (MY32 anti‐fast and NOQ7.5.4D anti‐slow myosin). Both species, *S*. *scincus* (Figure 8a,b) and *E*. *schneideri* (Figure 8c,d) exhibited the same pattern of immunochemical‐staining, with the muscle fibers incubated with anti‐fast myosin antibodies presenting three different colors (Figure 8a,c): dark brown (fast fibers), light brown (intermediate fibers), and blue (slow fibers). The slow fibers were negative and only stained blue after counterstaining with luxol blue. To confirm the specificity of the method, immunohistochemical staining was conducted systematically on the following section with an antibody raised against slow myosin. The pattern of DAB‐positive staining corresponded exactly to the slow muscle fibers that were negative for staining with the anti‐fast antibody (Figure 8 b vs. a and d vs. c). In contrast to the anti‐fast myosin antibody, the anti‐slow myosin antibodies did not allow differentiation of fast muscle fibers from intermediate fibers, and both were negative with this antibody.

#### 3.3.2 Muscle fiber typing

The relative areas occupied by each type of muscle fiber along the body were morphometrically compared between *S*. *scincus* and *E*. *schneideri*. In general, it appears that fast fibers predominated in both species, accounting for a mean of 62–63% of the total body muscle area. Intermediate fibers represented 25–29% of the total muscle area, and slow fibers were the least abundant, accounting for 7–11% of the total muscle area. The relative density of each of the fiber categories was compared between the two species using three Wilcoxon tests. The relative densities of fast and intermediate muscle fibers were significantly greater for *S*. *scincus*. Inversely, the relative area occupied by slow fibers was greater for *E*. *schneideri* (*p* < .01). The mean individual area of each fiber type (in cross section) was also compared between both species. The mean individual area of fast, intermediate, and slow fibers in cross section was, for all three, significantly higher for *E*. *schneideri* than *S*. *scincus*. The mean diameters of fast fibers in cross section were 52.8 and 61.6 μm for *S*. *scincus* and *E*. *schneideri*, respectively. The mean diameter of intermediate fibers was 59.9 μm for *S*. *scincus* and 72.7 μm for *E*. *schneideri*. For the slow fibers, the mean diameter values were 43.6 μm for *S*. *scincus* and 51.4 μm for *E*. *schneideri*. Additionally, a comparative study of the absolute area accounted for the three types of fibers was performed separately on the ventral (*M*. *rectus abdominus*) and the dorsal (*M*. *longissimus dorsi*) muscular layers of the lizards' bodies. No significant difference (Table 1) was observed in *S*. *scincus*, whereas two significant intraspecific differences were observed in *E*. *schneideri*. In the latter, the proportion of the fast fibers was higher in the *M*. *rectus abdominus*, and inversely the slow fibers in the *M*. *rectus abdominus* were less abundant than in the dorsal muscles (*M*. *longissimus dorsi*). Beside these intraspecific comparisons, we estimated the relative abundance of each fiber type for both species in the dorsal (*M*. *longissimus dorsi*) and ventral (*M*. *rectus abdominus*) muscles (Table 2). The results show that the general pattern is conserved, with a high abundance of fast fibers (60–62%), an intermediate abundance of intermediate fibers (24–30%), and a low abundance of slow fibers (9–14%). The individual area of each type of muscle fiber (in cross section) was also evaluated separately for the dorsal (*M*. *longissimus dorsi*) and ventral (*M*. *rectus abdominus*) muscles (Table 1). The fast fibers accounted for a greater area in *E*. *schneideri* than in *S*. *scincus*, but this significant difference was only observed between the ventral fibers of *E*. *schneideri* and both the dorsal and ventral fibers of *S*. *scincus*. The intermediate fibers accounted for a larger cross‐sectional area in *E*. *schneideri* than in *S*. *scincus*. These significant differences were observed between the dorsal muscles (*M*. *longissimus dorsi*) of *E*. *schneideri* and the intermediate muscle fibers of *S*. *scincus*, regardless of their localization. Finally, the mean individual area of slow muscle fibers exhibited by *E*. *schneideri* was also significantly greater than that for *S*. *scincus*, but only between the ventral region (*M*. *rectus abdominus*) of *E*. *schneideri* and the dorsal region (*M*. *longissimus dorsi*) of *S*. *scincus*.

**TABLE 1:** Results of the statistical tests performed on all the fiber typing data

Data	Statistical test	Groups compared	*p*‐Values (.05 threshold)
Total scanned surface fibers in μm^2^ (dorsal vs. ventral fibers)	Wilcoxon	Fast *S*. *scincus*	NS
		Intermediate *S*. *scincus*	NS
		Slow *S*. *scincus*	NS
		Fast *E*. *schneideri*	***p* < .05**
		Intermediate *E*. *schneideri*	NS
		Slow *E*. *schneideri*	***p* < .05**
Cross sectional area (dorsal vs. ventral fibers)	Kruskal–Wallis	Fast	*E*. *schneideri* vs. *S*. *scincus* ventral ***p* < .05**
		Intermediate	*E*. *schneideri* vs. *S*. *scincus* dorsal ***p* < .05**
		Slow	NS
Total scanned surface fibers in μm^2^ (body vs. tail fibers)	Wilcoxon	Fast *S*. *scincus*	NS
		Intermediate *S*. *scincus*	NS
		Slow *S*. *scincus*	***p* < .05**
		Fast *E*. *schneideri*	NS
		Intermediate *E*. *schneideri*	NS
		Slow *E*. *schneideri*	NS
Cross sectional area (body vs. tail fibers)	Kruskal–Wallis	Fast	*E*. *schneideri* vs. *S*. *scincus* body ***p* < .05**
			*E*. *schneideri* vs. *S*. *scincus* tail ***p* < .05**
		Intermediate	*E*. *schneideri* vs. *S*. *scincus* body ***p* < .05**
			*E*. *schneideri* vs. *S*. *scincus* tail ***p* < .05**
		Slow	*E*. *schneideri* vs. *S*. *scincus* body ***p* < .05**

*Note:* The bold *p*‐values indicate a statistically significant difference, inferior to the .05 threshold. The “NS” abbreviation indicates a nonstatistically significant difference. The Wilcoxon tests were used when two groups of fibers were compared (e.g., the dorsal and ventral fast fibers of *Scincus scincus*). The Kruskal–Wallis tests were used when at least three groups were compared (e.g., the dorsal and ventral fast fibers of *S*. *scincus* and *Eumeces schneideri*). For the Wilcoxon tests, all the comparisons values are indicated in the last column. For the two sets of cross‐sectional areas values analyzed by Kruskal–Wallis tests, only the most important comparisons are indicated in the last column.

**TABLE 2:** The relative proportion in % (mean values ± *SD*) of each muscle fiber type in function of dorsal or ventral localization for both species. The total corresponds to 100% of muscular fibers present in ventral or dorsal part of the body. These percentages were calculated from four microscopic fields (390 × 10^3^ μm^2^) taken at random in the dorsal and ventral part of the lizard body

Species			
*Eumeces schneideri*		*Scincus scincus*	
Dorsal			
Fast	60.34 ± 20.09	Fast	62.76 ± 23.77
Intermediate	24.67 ± 13.78	Intermediate	26.16 ± 16.85
Slow	14.99 ± 14.31	Slow	11.08 ± 12.03
Ventral			
Fast	66.60 ± 28.51	Fast	58.35 ± 23.23
Intermediate	24.27 ± 26.93	Intermediate	30.20 ± 18.00
Slow	9.13 ± 12.15	Slow	11.45 ± 13.8

Morphometric measurements were also performed in order to compare the morphology of the muscles in the tail versus those in the body (Table 1). The tail was sectioned transversely every centimeter along the tail starting from the backside of cloaca to the end of the tail. As shown in Figure 1, the tail of *S*. *scincus* is relatively shorter and more massive in comparison to that of *E*. *schneideri*. The density of each type of fiber observed in the tail was similar to those recorded from the trunk with the exception of slow fibers, which presented a statistically greater density in the body of *S*. *scincus*. As presented in Table 3, the general pattern of fiber types in the tail was similar to that described for the trunk, that is, it is characterized by a predominance of fast fibers. However, slow fibers were significantly more abundant in the body muscles of *S*. *scincus*, where they represent 9.73% of the total, versus 7.81% in the caudal region. The same tendency is evident for *E*. *schneideri*. The other intraspecific differences between the scanned areas are nonsignificant. Finally, the individual area of each type of muscle fiber (in cross section) was also compared for trunk and the tail of both species (Table 1). Numerous interspecific significant differences are evident for the three categories of muscle fibers. Independently of the body region, the individual area of muscle fibers was larger in *E*. *schneideri* than *S*. *scincus*.

**TABLE 3:** The relative proportion in % (mean values ± *SD*) of each muscle fiber type in function of corporal or caudal localization for both species. The total corresponds to 100% of muscular fibers present in body or tail part of the lizard. These percentages were calculated from eight microscopic fields (780 × 10^3^ μm^2^) taken at random in the body and tail, respectively

Species			
*Eumeces schneideri*		*Scincus scincus*	
Body			
Fast	64.56 ± 26.47	Fast	62.06 ± 25.34
Intermediate	23.40 ± 23.16	Intermediate	28.21 ± 20.57
Slow	12.04 ± 15.42	Slow	9.73 ± 11.43
Tail			
Fast	63.03 ± 27.05	Fast	61.25 ± 23.24
Intermediate	28.62 ± 23.15	Intermediate	30.94 ± 19.18
Slow	8.35 ± 9.97	Slow	7.81 ± 13.01

## 4 DISCUSSION

### 4.1 Integument histology

The general histological pattern of the integument corresponds to the classical description of Lepidosaurian skin, with an epidermis composed of an outer *stratum corneum* that is heavily keratinized and capped by external microornamented Oberhäutchen layer, an intermediate hard keratin‐β layer, and an inner soft keratin‐α layer. These three layers form scales overlying two layers of keratinocytes, that is, the *stratum granulosum* and the *stratum germinativum* (Alibardi, 1997). The outermost layer of epidermis, the Oberhäutchen strata, is microornamented. This has been extensively studied and has been correlated with the ecology of certain lizards, such as lacertids (Arnold, 2002). It seems that this correlation must be considered carefully, the choice between ecological and phylogenetic hypothesis being in conflict, as explained in Gower (2003). The microornamentation could be linked to the physical environment and/or genetic background. The dermis of the two studied species contains osteoderms, which is characteristic of some lepidosaurians, such Scincidae, Anguidae, and Helodermatidae. Within Scincoidea, the Scincidae have a specific pattern of osteoderms referred as “compound osteoderms” (Vickaryous & Sire, 2009). The structures observed for both species, *S*. *scincus* and *E*. *schneideri*, correspond to this description. We should add that all osteoderms of these two species seem to possess a capping tissue. This tissue seems acellular and is overlapping a collagen dense bone tissue. This structure may be similar to the so‐called “osteodermine” tissue described in certain species such as Helodermatids (Kirby et al., 2020), but additional analysis might be necessary to solve this question. The melanocytes are located just under the *stratum germinativum* on the upper side of the dermis (*stratum laxum*). The dermis of both species presents the common characteristics of the dermis of squamates (Bellairs, 1969; Rutland, Cigler, & Kubale, 2019), being mainly composed of fibrous collagen. The deep dermis is separated from the underlying body wall muscles by a thin hypodermis, consisting of adipocytes. Histological staining allowed us to identify holocrine glands that appear to correspond to structures described for the skink, *Lampropholis guichenoti* (Alibardi & Thompson, 1999). For these authors, the presence of “sebaceous‐like secretory cells” at the overlapping space between macroscopic folds of the skin, producing a lipid like material, is linked to a possible friction reduction role. The structures observed for both, *S*. *scincus* and *E*. *schneideri*, seem to be similar, and we suspect that their role is quite similar, considering the fact that these structures are positioned in the hinge between tegument folds. The product of these secretory cells could protect against the accumulation of sand grains in the hinges. Using SEM at the surface of the scales, we have documented a geometrical pattern composed of microridges, with those for the species *E*. *schneideri* being more spaced, and the microornamentation pattern of *S*. *scincus* being smoother. Similarities between the dorsal and ventral patterns of *S*. *scincus* have recently been observed by Allam et al. (2017). This study also exhibited the same results concerning the rough dorsal pattern of *E*. *schneideri* scales, and the more corrugated pattern of its ventral scales. Here, we have also added a quantitative distinction between the two skink species, *S*. *scincus*, exposing a generally smoother Oberhäutchen layer with the microridges being more spaced. This pattern could be linked to environmental constraints. As exposed by Allam et al. (2017), the microstructures of these two Scincidae could be related to their microhabitat, *S*. *scincus* living much more in sand dunes and presenting a less frictional surface due to its smoother scales. The results that we have obtained for *S*. *scincus* and *E*. *schneideri* may correspond to this ecological hypothesis.

### 4.2 Epidermal dividing cells

The greater number of proliferating cells at the level of the epidermis of *E*. *schneideri* may be an indication that this species possesses less resistant skin than *S*. *scincus*. As opposed to mammals that have a continuous renewal of their epidermal cells, lepidosaurians undergo a cyclical process. This epidermal generation is a synchronized process taking place over the entire body surface (Maderson, Rabinowitz, Tandler, & Alibardi, 1998). It is relevant to highlight that squamates have a discontinuously generated epidermis following a resting phase, during which just a few new cells are generated (Downing & Roth, 1974). It should be noted that the two skinks studied here shed their skin not in one piece, like snakes, but in small flakes (Swadzba, Maslak, & Rupik, 2009). We also need to highlight the fact that during a resting phase, little or no proliferation occurs at the epidermal level. Considering this, and in relation to the high number of labeled PCNA epidermal cells obtained for both species, we can conclude that the sacrificed individuals of both species were in an active phase of cell proliferation. However, we cannot be sure that all individuals of both species were at exactly the same moment of their shedding cycle, thus differences in labeled cells may be affected by differences in the physiological state of the molt cycle. The duration of this phase is about 14 days in all species of lepidosaurians that have been studied so far (Alibardi, Maurizii, & Taddei, 2001). We must also consider that the renewal phase represents only a short time period as compared to the resting time, especially during fall and winter, as observed for northern species of squamates (Alibardi, Maurizii, & Taddei, 2000). We have found no studies so far on the epidermal renewal among psammophilous skinks, or in lepidosaurians. Our results seem to indicate that the phases of cell proliferation could be more frequent in these skinks and/or that the rest phases could be shorter, which would allow faster regeneration of the epidermis exposed to strong and constant abrasion of the substrate. Supposing that the studied specimens were labeled at the same moment of the regeneration phase, the higher proliferating rate found in *E*. *schneideri* could indicate that this species responds to abrasion with a high rate of epidermal regeneration while *S*. *scincus* would resist abrasion with a smoother and more resistant epidermal scales which requires a slower turnover. It is also possible that the time of maturation and progression of the keratinocytes through the superficial layers of the epidermis differs between the two species. We must also take into account that the PCNA is also implicated in DNA reparation, and that it does not show only the proliferating cells; the estimated proliferation may therefore be affected. However, during the repair process the quantity of enzyme (PCNA) present in the nucleus is low compared to the high quantity present during the S phase. In general, the low quantity of PCNA present during a DNA‐repair process is below the detection threshold by immunocytochemical methods. Therefore, we can consider that the nuclei that we have highlighted correspond overwhelmingly to cells engaged in the process of mitotic division.

*S*. *scincus* possesses remarkable skin properties, such as a low friction angle that seems to allow this species to optimally resist the constant sand abrasion to which it is subjected (Baumgartner et al., 2007). The resistant properties of its skin are seemingly due to its chemical properties, promoting low adhesion, which prevents the formation of van der Waals bonds between it and the substratum (Staudt, Böhme, & Baumgartner, 2012), more precisely to the presence of neutral glycans (Vihar, Hanisch, & Baumgartner, 2016). Considering this, it seems that *S*. *scincus* does not need a high turnover of its epidermal cells in contrast to *E*. *schneideri*. Finally, we must add that skin thickness does not support the hypothesis of a thicker epidermis for a species like *S*. *scincus*, which is potentially more frequently exposed to sand abrasion. Actually, the absolute thickness was higher in *E*. *schneideri*. This parameter should be compared to other psammophilous squamates to test the hypothesis that abrasion and skin relative thickness are correlated.

### 4.3 Muscle fiber pattern

The muscular system is supposedly designed to meet functional morphological requirements involved in motion, and has long been the subject of studies, such as that of Putnam, Gleeson, and Bennett (1980). This study exhibited the link between the fast twitch glycolytic fibers of the locomotory muscles of *Dipsosaurus dorsalis* (Iguanidae) and its fast locomotor behavior. A more recent study has shown the differences in muscle fiber composition among some phrynosomatids (Bonine, Gleeson, & Garland Jr, 2001). This clearly showed a relationship between the phylogeny of three subclades of this family and the relative proportion of muscle fiber typing. More precisely, the “sand lizards” subclade had a high proportion of fast twitch glycolytic fibers compared to the “horned lizards” subclade, which had a higher proportion of fast twitch oxidative‐glycolytic fibers (which correspond to the intermediate fibers). These differences, phylogenetically marked, are coherent with the locomotor performance of these two subclades, the “sand lizards” being fast sprinters and the “horned lizards” being much slower. A study by Kohlsdorf et al. (2004) on *Tropidurus* (Tropiduridae) highlighted the fact that the absolute performance, indeed the sprint speed, of the sand dune species *Tropidurus psamonastes*, was well predicted by the relative content of glycolytic fibers in the *m*. *iliofibularis* (an important hind limb muscle). Skeletal muscle present high plasticity, adapting to a variety of external stimuli (Booth & Thomason, 1991; Hawley, 2002; Luna, Daikoku, & Ono, 2015; Scales, King, & Butler, 2009). This includes the force necessary to elicit the movement that depends on various factors, such as body weight, the mechanical resistance of the substrate, the level of contractile activity (e.g., the necessity of an explosive strength of muscles or inversely the endurance training), the protein and energetic resources availability in food, and the prevailing environmental conditions (e.g., thermal stress, the oxygen level, etc.). Phenotypic plasticity is observed in all clades of vertebrates (Schiaffino & Reggiani, 1996, 2011). However, there is a large variation of adaptability among species that depend on genetic predispositions specific to each species (Flück & Hoppeler, 2003). The typing of muscle fibers in psammophilous Scincidae is so far entirely unexplored. In the present study, we have highlighted a clear predominance of fast muscle fibers in both studied species. This kind of muscle fiber represents more than 60% of the muscular mass. This high proportion of fast muscles allows powerful, fast, and short‐duration movements in a medium that offers high mechanical strength. Some cinematic studies analyzing the locomotion of *S*. *scincus* in granular media via X‐Rays (Maladen et al., 2009; Maladen, Ding, et al., 2011; Maladen, Umbanhowar, et al., 2011) or NMR imaging (Baumgartner et al., 2008) have reported a velocity of 30 cm/s on distances of several meters (Rechenberg, 2009). This behavior, adopted by the sandfish, allows them to bury themselves quickly to escape predators (Arnold, 1995) and to adapt their body temperature that could decline with the burial depth. This type of behavior is also present in other reptiles living in the same biotope of aeolian sand, such as the Kenyan sand boa (*Gongylophis colubrinus*) (Al‐Sadoon & Al‐Otaibi, 2014; Al‐Sadoon, Al‐Otaibi, & Oraby, 2013; Klein et al., 2010), the sepsoid skink (*Chalcides sepsoides*) (Carranza, Arnold, Geniez, Roca, & Mateo, 2008), and the Berber skink (*E*. *schneideri)* (Perera, Sampaio, Costa, Salvi, & Harris, 2012; Warburg, 1978).

The results of our morphometric analysis showed that fast fibers have a much higher diameter than the slow fibers, and, supposing that fiber diameter is indicative of force output, are able to generate a more powerful muscular force which could facilitate movement in this granular medium, which offers a high mechanical resistance by friction. However, we could speculate that the high proportion of fast fibers observed in both species represents an adaptation to the anaerobic conditions in the fine granular substrate. Indeed, fast muscles essentially work by anaerobic glycolysis and therefore are well suited to a low oxygen environment (Kanatous et al., 2008). When the animal is buried in the sand it can use its low oxygen reserve to supply its vital organs (brain, heart, etc.) since the oxygen consumption in the glycolytic fast muscles is low, as it is also the case for mammalians (Shero, Andrews, Lestyk, & Burns, 2012). We suggest that the mechanical resistance and the constant hypoxia, parameters that are not mutually exclusive, explain the higher relative proportion of fast glycolytic fibers in *S*. *scincus*. The second category of fiber in terms of abundance in both studied species is the intermediate fibers. This type of muscle fiber allows the mode of energy production to be adapted according to the blood oxygenation rate (Mandic, Lau, Nijjar, & Richards, 2008; Westerblad, Bruton, & Katz, 2010). So, they will essentially produce their ATP by anaerobic glycolysis when the lizard is buried in sand, but will switch to oxidative phosphorylation when the animal is moving in the open air and run over greater distances. Indeed, these fibers are much more resistant to fatigue than fast fibers (Westerblad et al., 2010). It is noteworthy that the results we have obtained for the total scanned area of intermediate fibers showed a significantly higher relative content for *S*. *scincus*. This result could be explained by the need for a more adaptive muscular system due to the different ecological behavior of the sandfish (Hetherington, 1989). This could be linked to the alternation between resting time buried deeply in the sand and intense movements during feeding time at a more superficial level. Both species have a low proportion of slow fibers (less than 12%) and this proportion declines in the tail compared to the body. For *S*. *scincus*, this significant difference could be explained by the short and dense morphology of the tail and its involvement in motion. The lower proportion of slow fibers seen in the tail of *S*. *scincus* is probably related to its locomotory behavior, implicating impressive sinusoidal bending movements during which the tail seems to follow the rest of the body (Maladen et al., 2009). It could be argued that its tail has to perform fast and powerful movements in mechanical and anaerobic harsh conditions. Consequently, the short, thick sandfish tail is rich in fast glycolytic fibers and acts as a powerful “caudal fin” when the animal swims in the sand. In contrast, the tail of *E*. *schneideri* is much longer and thinner, and serves mainly as a pendulum when the animal moves on the surface, climbs rocks, or jumps from one twig to another in the semidesert vegetation, which constitutes their biotope of predilection. The tail of *E*. *schneideri* is also able to divert the attention of predators by autotomy while squirming for a number of minutes. However, after autotomy, the regenerated tail is shorter, and the slow fibers are replaced by fast and intermediate fibers, as has been reported for other lizard species (Alibardi, 2015). The anatomical differences, and the different functions, assumed by the tail in the two species studied may explain the variations in the proportion of muscle fiber populations. A comparative behavioral study would be interesting to investigate the tail movements in each species. However, the importance of the relative proportion of the “slow oxidative” fibers seems less clear, as discussed by Bonine et al. (2001). Indeed, this kind of fiber may be less dependent on selective pressure and is perhaps subjected to neutral selection (Scales et al., 2009). Another important factor to consider is temperature. As noted by Jayne and Daggy (2000), lizards, as ectothermic animals, encounter a wide range of temperatures, which can affect their locomotor performance and muscle function. More precisely, ectothermic vertebrates increase the recruitment of faster muscle fibers at lower temperatures (Rome, Choi, Lutz, & Sosnicki, 1992). The importance of temperature for our two scincids, especially for *S*. *scincus*, may be moderate, considering the fact that the sand swimming behavior allows a more suitable place to regulate body temperature to be chosen rapidly. The importance of this factor remains to be investigated, but we suspect that its impact on muscular patterns might be limited.

Taken together, the results of our study allow us to add new insights to the comprehension of the remarkable morphological structures of the studied species. However, a broader comparison with other non‐psammophilous species of Scincoidea superfamily is needed to determine whether the various features reported here are similar as a result of convergence, parallelism, or shared ancestry. As noted in a recent study by Wu et al. (2018), which has suggested a lesser importance of the sandfish skin's tribological properties, there was a need to precisely understand the other mechanisms explaining the adaptation of *S*. *scincus* to its sandy substrate. The relative importance of the integument (and more particularly of the dermis) combined with the muscle system may be greater than previously thought, and could at least act in combination with the superficial epidermal properties. The integument of skinks, containing many layers of osteoderms, allows them to ally a “natural armor” while maintaining a relative flexibility required for locomotion (Chintapalli, Mirkhalaf, Dastjerdi, & Barthelat, 2014). Beside the fact that osteoderms probably play a role in locomotion, as reiterated in the study of Burns, Vickaryous, and Currie (2013) performed on crocodilians, there is a lack of knowledge about the link between osteoderms and locomotion for specific clades like the psammophilous scincids. Moreover, there are almost no studies on the other hypothetical roles played by osteoderms, like thermoregulation (Broeckhoven, du Plessis, & Hui, 2017). We postulate that the thick strata of osteoderms present in *S*. *scincus* (and to a lesser degree of *E*. *schneideri*) could act like an “exoskeleton” combining the flexibility and resistance of the integument and offering a point of support and a mechanical relay to the underlying muscle stratum, mostly composed of fast fibers. This combination of fast muscles and osteodermic strata could allow rapid movements in the sandy substrate with high mechanical strength and poor oxygen levels.

## CONFLICT OF INTEREST

Both authors declare no conflicts of interest.

## AUTHOR CONTRIBUTIONS

**Jérôme Canei**: Conceptualization, investigation, formal analysis, methodology, writing‐original draft, writing‐review, and editing. **Denis Nonclercq**: Conceptualization, supervision, project administration, resources, writing‐review, and editing.

## Supporting information


**Figure S1**_SuppInfo.pdf. Schematic transversal views of the body A and tail B parts of both species. A: The body muscles targeted (delimited by a red square) were the *longissimus dorsi* for the dorsal parts, and the *rectus abdominus* for the ventral parts. The morphology of these muscles was illustrated at low magnification on the right‐side microscopic fields. B: The muscular bundles located beyond the cloaca were considered as “tail muscles.”. The precise nomenclature of the tail muscles is not clearly specified in the literature. Consequently, we have chosen to consider all the muscular fibers surrounding the tail vertebras as illustrated by red squares on scheme. The right‐side picture illustrates the histology of these tail muscular bundles.

**Figure S2**_SuppInfo.pdf. High magnification of the ventral holocrine glands of *Scincus scincus* (A) and *Eumeces schneideri* (B). The stratified glandular cells present a progressive accumulation of lipidic droplets in their cytoplasm (arrows). These cells death and release their secretions (stained in orange) in spaces between macroscopic folds of the skin.

**Table S1**_SuppInfo.pdf. Mean values (± SD) of the length between consecutive microridges of *Scincus scincus* and *Eumeces schneideri*. The dorsal and ventral values are in μm. The mean height values (± SD) of these microridges is also in μm. Only the dorsal ones were considered, the margin of error being too important for the ventral ones.

## Data Availability

The data that support the findings of this study are available on request from the corresponding author.
