# Supplementary material for: Morphological study of the integument and corporal skeletal muscles of two psammophilous members of Scincidae (Scincus scincus and Eumeces schneideri)
Source: J Morphol. 2020 Nov 9;282(2):230–46. doi: 10.1002/jmor.21298 (PMC7839682; doi:10.1002/jmor.21298)
Supplement: Supplementary file 1 — Figure S1_SuppInfo.pdf. Schematic transversal views of the body A and tail B parts of both species. A: The body muscles targeted (delimited by a red square) were the longissimus dorsi for the dorsal parts, and the rectus abdominus for the ventral parts. The morphology of these muscles was illustrated at low magnification on the right‐side microscopic fields. B: The muscular bundles located beyond the cloaca were considered as “tail muscles.”. The precise nomenclature of the tail muscles is not clearly specified in the literature. Consequently, we have chosen to consider all the muscular fibers surrounding the tail vertebras as illustrated by red squares on scheme. The right‐side picture illustrates the histology of these tail muscular bundles. [file JMOR-282-230-s001.docx]

**
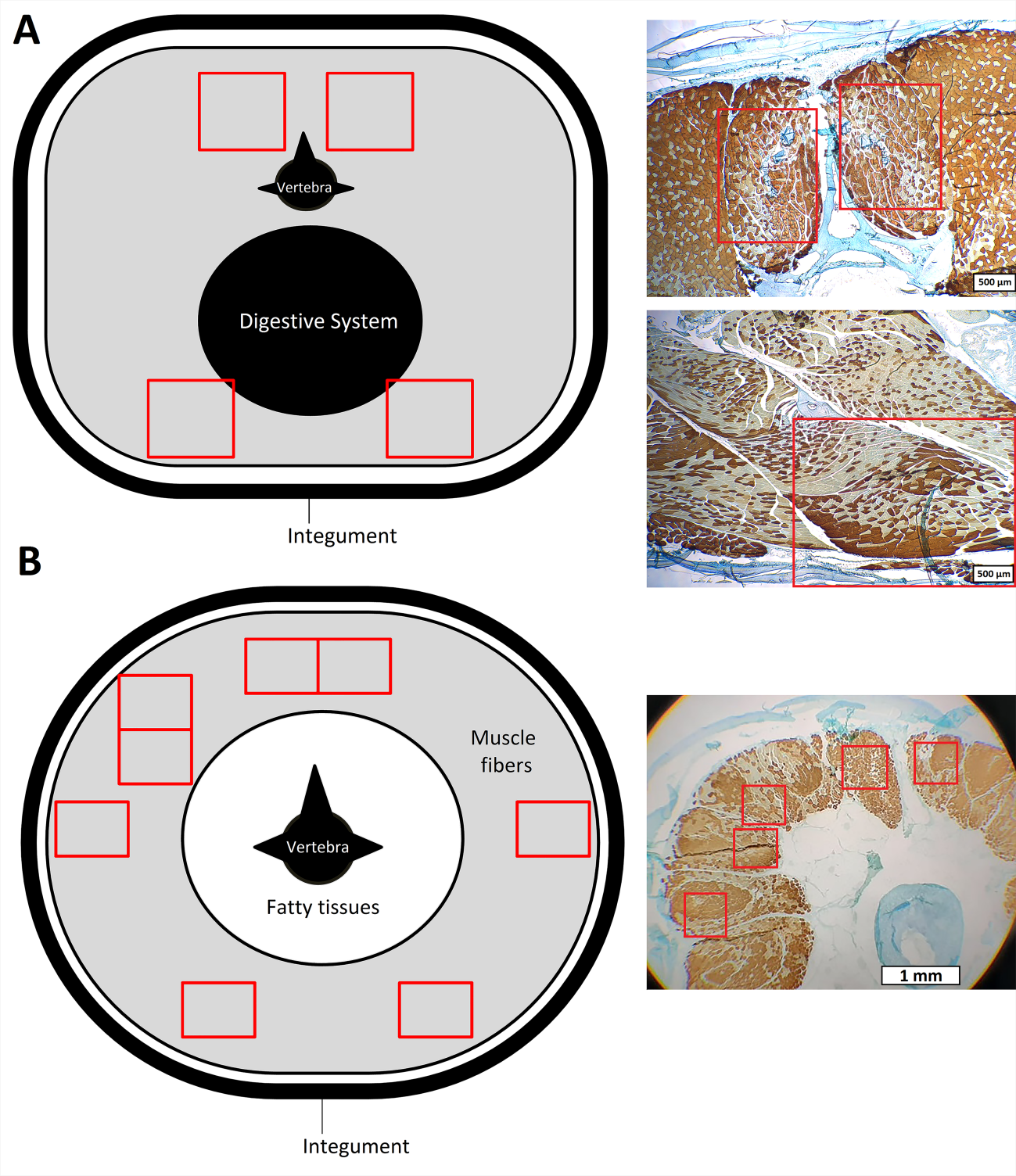
**

**Figure A1**_SuppInfo.pdf**.** Schematic transversal views of the body A and tail B parts of both species. A: The body muscles targeted (delimited by a red square) were the *longissimus dorsi* for the dorsal parts, and the *rectus abdominus* for the ventral parts. The morphology of these muscles was illustrated at low magnification on the right-side microscopic fields. B: The muscular bundles located beyond the cloaca were considered as "tail muscles". The precise nomenclature of the tail muscles is not clearly specified in the literature. Consequently, we have chosen to consider all the muscular fibers surrounding the tail vertebras as illustrated by red squares on scheme. The right-side picture illustrates the histology of these tail muscular bundles.
