# Supplementary material for: Morphological study of the integument and corporal skeletal muscles of two psammophilous members of Scincidae (Scincus scincus and Eumeces schneideri)
Source: J Morphol. 2020 Nov 9;282(2):230–46. doi: 10.1002/jmor.21298 (PMC7839682; doi:10.1002/jmor.21298)
Supplement: Supplementary file 2 — Figure S2_SuppInfo.pdf. High magnification of the ventral holocrine glands of Scincus scincus (A) and Eumeces schneideri (B). The stratified glandular cells present a progressive accumulation of lipidic droplets in their cytoplasm (arrows). These cells death and release their secretions (stained in orange) in spaces between macroscopic folds of the skin. [file JMOR-282-230-s002.docx]

**
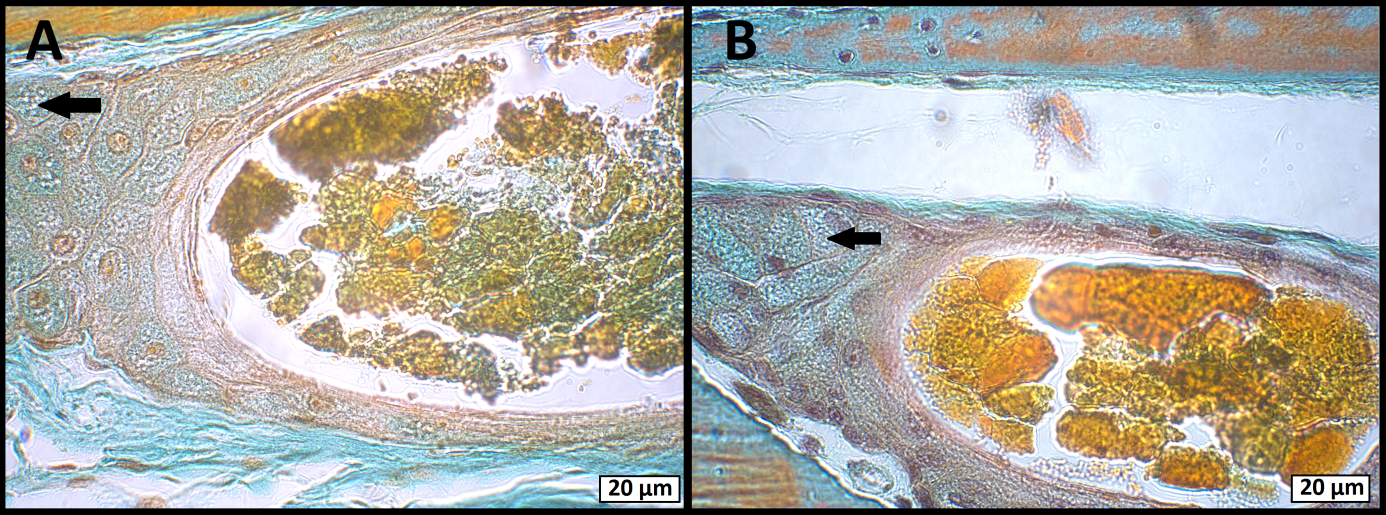
**

**Figure A2**_SuppInfo.pdf**.** High magnification of the ventral holocrine glands of *Scincus scincus* (A) and *Eumeces schneideri* (B). The stratified glandular cells present a progressive accumulation of lipidic droplets in their cytoplasm (arrows). These cells death and release their secretions (stained in orange) in spaces between macroscopic folds of the skin.
