# Supplementary material for: Morphological study of the integument and corporal skeletal muscles of two psammophilous members of Scincidae (Scincus scincus and Eumeces schneideri)
Source: J Morphol. 2020 Nov 9;282(2):230–46. doi: 10.1002/jmor.21298 (PMC7839682; doi:10.1002/jmor.21298)
Supplement: Supplementary file 3 — Table S1_SuppInfo.pdf. Mean values (± SD) of the length between consecutive microridges of Scincus scincus and Eumeces schneideri. The dorsal and ventral values are in μm. The mean height values (± SD) of these microridges is also in μm. Only the dorsal ones were considered, the margin of error being too important for the ventral ones. [file JMOR-282-230-s003.docx]

**Table A1**_SuppInfo.pdf**.** Mean values **(**± SD) of the length between consecutive microridges of *Scincus scincus* and *Eumeces schneideri*. The dorsal and ventral values are in µm. The mean height values **(**± SD) of these microridges is also in µm. Only the dorsal ones were considered, the margin of error being too important for the ventral ones.

| **Species** | |
| --- | --- |
| ***Eumeces schneideri*** | ***Scincus scincus*** |
| Dorsal length | |
| 2.15 **±** 0.4 | 7.44 **±** 1.39 |
| Ventral length | |
| 5.32 **±** 0.57 | 11.32 **±** 1.96 |
| ***Eumeces schneideri*** | ***Scincus scincus*** |
| Height | |
| 0.423 ± 0.06 | 0.408 ± 0.10 |
